# Temporal and Embryonic Lineage-Dependent Regulation of Human Vascular SMC Development by NOTCH3

**DOI:** 10.1089/scd.2014.0520

**Published:** 2014-12-24

**Authors:** Alessandra Granata, William G. Bernard, Ning Zhao, John Mccafferty, Brenda Lilly, Sanjay Sinha

**Affiliations:** ^1^ Anne Mclaren Laboratory for Regenerative Medicine, Wellcome Trust-Medical Research Council, Cambridge Stem Cell Institute, University of Cambridge, Cambridge, United Kingdom.; ^2^ Division of Cardiovascular Medicine, University of Cambridge, Cambridge, United Kingdom.; ^3^ Department of Pediatrics, Center for Cardiovascular and Pulmonary Research, Nationwide Children's Research Institute, The Ohio State University, Columbus, Ohio.; ^4^ Iontas Ltd., Cambridge, United Kingdom.

## Abstract

Vascular smooth muscle cells (SMCs), which arise from multiple embryonic progenitors, have unique lineage-specific properties and this diversity may contribute to spatial patterns of vascular diseases. We developed in vitro methods to generate distinct vascular SMC subtypes from human pluripotent stem cells, allowing us to explore their intrinsic differences and the mechanisms involved in SMC development. Since Notch signaling is thought to be one of the several key regulators of SMC differentiation and function, we profiled the expression of Notch receptors, ligands, and downstream elements during the development of origin-specific SMC subtypes. *NOTCH3* expression in our in vitro model varied in a lineage- and developmental stage-specific manner so that the highest expression in mature SMCs was in those derived from paraxial mesoderm (PM). This pattern was consistent with the high expression level of NOTCH3 observed in the 8–9 week human fetal descending aorta, which is populated by SMCs of PM origin. Silencing *NOTCH3* in mature SMCs in vitro reduced SMC markers in cells of PM origin preferentially. Conversely, during early development, *NOTCH3* was highly expressed in vitro in SMCs of neuroectoderm (NE) origin. Inhibition of NOTCH3 in early development resulted in a significant downregulation of specific SMC markers exclusively in the NE lineage. Corresponding to this prediction, the Notch3-null mouse showed reduced expression of Acta2 in the neural crest-derived SMCs of the aortic arch. Thus, Notch3 signaling emerges as one of the key regulators of vascular SMC differentiation and maturation in vitro and in vivo in a lineage- and temporal-dependent manner.

## Introduction

A primary role of mature vascular smooth muscle cells (SMCs) is to maintain vascular tone and regulate blood pressure and blood flow distribution through constriction and dilation of the vessels [1]. In adult organisms, SMCs show extensive differences in morphology and function and these may depend on their position in the vascular bed, the organ context, and their embryological origin. Furthermore, adult vascular SMCs still maintain a remarkable phenotypic plasticity by undergoing profound and reversible changes, such as proliferation, migration, and downregulation of contractile genes, following vascular injury or disease [2].

Importantly, the origin of SMCs within the major arteries of the trunk is complex with contributions from several independent cell lineages. In particular, SMCs of the developing aortic arch, ductus arteriosus, pulmonary trunk, and the proximal regions of the major aortic arch branches derive from the cardiac neural crest [3–5], while the second heart field (lateral mesoderm [LM] origin) gives rise to SMCs at the root of the aorta, and the somites [paraxial mesoderm (PM) origin] generate SMCs of the descending aorta [6,7].

It is known that vascular SMC subtypes show lineage-specific differences in growth, gene expression, and functional properties [8], and these differences may contribute to site-specific patterns of vascular diseases, such as aortic aneurysm.

To better comprehend the intrinsic differences between origin-specific SMC lineages and to study the role that specific signaling pathways play in each lineage, our group has generated an in vitro model for lineage-specific vascular SMCs originating from human pluripotent stem cells [9]. Origin-specific SMCs derived using this system replicate many of the known differences between SMCs of different lineages, such as response to TGF-β1 or requirement for MKL/myocardin-like 2 (MKL2) in development [9]. Moreover, this in vitro model correctly predicted the proteolytic properties of different tissue-derived vascular SMCs.

For the studies in the present article, we have focused our attention on the Notch pathway, which is known to play a key role in cardiovascular development, vascular repair of injury, and vascular pathology in humans [10,11]. Vascular SMCs express multiple Notch receptors, which are known to regulate smooth muscle differentiation, recruitment to growing vessels, and maturation. However, little is known about the involvement of distinct Notch family members in SMC lineages with different embryological origins.

Notch receptors are type-1 transmembrane proteins, which are activated upon interaction with two families of ligands: Jagged (JAG) or Delta-like (DLL) [12,13]. In mammals, the expression of four Notch receptors (NOTCH1, NOTCH2, NOTCH3, and NOTCH4) and five Jagged or Delta-like ligands (JAG1, JAG2, DLL1, DLL3, and DLL4) varies among cell types and changes in response to environmental cues within the surrounding tissue. Upon ligand interaction, Notch receptors undergo cleavage events and the soluble Notch intracellular domain (NICD) is released and translocates to the nucleus, where it forms an active transcriptional complex with the DNA-binding protein CSL (CBF-1/RBP-Jκ, Su(H), and Lag-1). This complex regulates downstream gene expression, including members of the HES (hairy/enhancer of split) and HRT (Hairy-related, also referred to as HEY, HERP, HESR, CHF) families [14].

Studies investigating the function of Notch signaling in vascular SMCs have shown contradictory results, describing both antidifferentiation [15,16] and prodifferentiation functions for Notch [17–20]. These contradictory results may be due to the context-dependent nature of potential inhibitory feedback by HRT family members and tight spatiotemporal regulation of Notch components during development. To help resolve this controversy, additional studies are essential, which aim to identify the expression pattern of different Notch ligands and receptors, the specificity of the ligands to the Notch receptors, and activation of downstream targets during SMC development and in the mature state. Importantly, it has been proposed that vascular SMCs of diverse embryonic origins have unique regulatory mechanisms of differentiation; therefore, it is important to examine the expression of Notch pathway components and their potential role in each distinct SMC subtype.

In this study, we show that expression of Notch receptors, in particular *NOTCH3*, ligands, and effectors varies during the development of origin-specific vascular SMC lineages in a lineage- and time-dependent manner. In addition, we demonstrate that specific inhibition of NOTCH3 causes downregulation of SMC markers in both paraxial mesoderm-derived SMCs (PM-SMCs) and neuroectoderm-derived SMCs (NE-SMCs) when the cells are fully differentiated, but only in SMCs of neuroectoderm (NE) origin at an early stage of development. Importantly, we then validate our novel in vitro findings using human fetal aortic tissues and a Notch3-null mouse model, suggesting that our human embryonic stem cell (hESC)-derived system closely mimics vascular SMC development in vivo.

## Materials and Methods

### Cell culture

Human H9 ESCs (passages 65–85) were cultured in a chemically defined medium (CDM) as previously described [21]. Human induced pluripotent stem cells (hiPSCs) were obtained from the Cambridge Biomedical Research Centre iPSC Core Facility and grown on irradiated mouse feeders in typical DMEM/F12 medium containing 20% knockout serum replacement (Gibco) and 4 ng/mL FGF-2 (R&D System). hESCs and hiPSCs were differentiated into the three intermediate populations as described in Cheung et al. 2012 [9]. After obtaining the intermediate populations, cells were trypsinized and cultured in SMC differentiation medium CDM containing PDGF-BB (10 ng/mL, PeproTech) and TGF-β1 (2 ng/mL, PeproTech) for at least 12 days. For long-term cultures, SMCs were grown in MEM medium (M5650; Sigma) containing 10% fetal bovine serum (FBS, F7524; Sigma) for up to 30 days.

### Quantitative real-time polymerase chain reaction

Developing SMCs were harvested at different time points before and during PDGF-BB and TGF-β1 treatment, and mature SMCs were harvested after 30 days of serum-contained medium culture. Whole heart and aorta were harvested from 8- to 9-week-old human fetal tissue (*n*=2; REC 96/085). obtained following therapeutic pregnancy interruption performed at Cambridge University Hospitals NHS Foundation Trust with ethical approval and informed consent. Aortic tissue was subsectioned into ascending aorta (AA), descending thoracic aorta (DTA), descending abdominal aorta (DAA), and pulmonary artery (PA) tissue. Tissues were homogenized in RLT buffer (Qiagen) utilizing Lysing Matrix D-Tubes (Anachem) and a Bio 101 FastPrep FP120 (Savant). Total RNA was extracted with the RNeasy Mini kit according to the manufacturer's instructions (Qiagen). cDNA was synthesized from 250 ng of RNA using the Maxima First Strand cDNA Synthesis Kit (Thermo Scientific). Quantitative real-time polymerase chain reaction (qRT-PCR) mixtures were prepared with SYBR GreenER PCR Super Mix (Invitrogen) and the reactions were performed in technical duplicates with the Rotor-gene 6000 system (Corbett Life Science), using the Quantitation–comparative CT settings. Primer sequences are listed in Supplementary Table S1 (Supplementary Data are available online at www.liebertpub.com/scd).

### Flow cytometry

For flow cytometry of NOTCH3, SMCs were harvested at the developing stage of day 12 for LM- and PM-SMCs and day 14 for NE-SMCs and at the mature stage after 30 days of serum-contained medium culture. SMCs transfected once or twice with specific Notch3 siRNA or scrambled siRNA were harvested 48 h after transfection. SMCs were fixed using the Cytofix/Cytoperm Fixation/Permeabilization kit (BD Biosciences) and stained according to the kit manual. Fixed SMCs were stained with rabbit polyclonal anti-NOTCH3 antibody 1:200 (ab23426; Abcam) for 1 h at 4°C, followed by two washes with the Cytowash buffer, and then incubated with a goat anti-rabbit Alexa Fluor 488 antibody for 30 s at 4°C, followed by two washes with the Cytowash buffer. Rabbit IgG isotope control (Sigma) was used. Cells were then resuspended in phosphate-buffered saline (PBS) and measured with Cyan ADP cell analyzer. Flow cytometric data were analyzed with FlowJo vX software.

### Cryosectioning and Immunohistochemistry

Whole aortas dissected from 8- to 9-week-old human fetal tissue (*n*=3) were fixed in 4% paraformaldehyde in PBS for 4–5 h at 4°C. Each sample was cryoprotected by consecutive incubation in 25% and 50% sucrose in PBS overnight. Then, the tissue was infiltrated with OCT medium, reoriented inside cryostat molds, and snap-frozen in liquid nitrogen. Embedded tissue was sectioned at 10 μm with a Cryostar NX70 cryostat (Thermo Scientific). Individual tissue sections were transferred to Superfrost Plus microscope slides (Fisher Scientific) and stored at −20°C. Tissue sections were rehydrated in PBS, followed by incubation with blocking solution [10% FBS and 1% bovine serum albumin (BSA) in PBS] for 1 h at room temperature. Serial sections were then incubated with primary antibodies, polyclonal rabbit anti-NOTCH3 1:200 and monoclonal antismooth muscle α-actin, ACTA2 1:400 (F3777; Sigma), overnight at 4°C. The sections were then washed in PBS and stained with secondary goat anti-mouse Alexa Fluor 568 and goat anti-rabbit Alexa Fluor 488 antibodies (Invitrogen Molecular Probes) for 1 h at room temperature. After PBS washing, the slides were mounted with aqueous mounting medium (VectaMount; Vector Laboratories) and coverslipped.

### Human aorta staining quantification

Images were acquired using 5X objective on a Zeiss Axiovert 200M epifluorescence microscope and 20X objective on a Zeiss Observer Z1 LSM 700 confocal microscope (three images of each aortic area for three different aortas were examined). All the images were collected with the same setting. Fluorescent images were analyzed with ImageJ software. The intensity of Notch3 staining in human sections was normalized to the one of DTA on the same section to correct for intersample variations in staining intensity.

### siRNA gene silencing and NOTCH3 blocking Ab treatment

Notch3 knockdown was carried out using GeneSolution siRNA (Qiagen). A nonspecific siRNA (AllStars Negative control; Qiagen) was used as a negative control. hESC-derived SMCs were transfected with siRNA (40 nm for one well of six-well plate) using DharmaFECT I transfection reagent (Thermo Scientific Dharmacon). The transfection was repeated after 2 days and the mRNA and protein analyses were performed after 24 and 48 h, respectively. The efficiency of *NOTCH3* knockdown by siRNA was ∼70%, and the siRNA exhibited maximum gene knockdown effectiveness after the second transfection (Supplementary Fig. S5).

A blocking antibody specific for NOTCH3 (AbN3; 10 ng/μL) given by Dr McCafferty [22] and a control anti-Desmin antibody (AbDesmin;10 ng/μL) were added to the medium of intermediate populations of LM and PM at day 5 and NE at day 7. The treatment was repeated after 3 days and the cells were harvested at day 7 and analyzed by qRT-PCR.

### Western blot

Western blot analysis was performed as previously described [9]. Primary antibodies used include polyclonal rabbit anti-Notch3 1:1,000 (ab23426; Abcam), monoclonal anti-smooth muscle α-Actin, 1:400 (DAKO), Calponin, 1:5,000 (CNN1, C2687; Sigma), and anti-β-actin 1:5,000 (A1978; Sigma).

### Mouse aortic staining and quantification

The *Notch3^−/−^* mouse strain was provided by Dr. Thomas Gridley [23]. Studies on these mice were approved by the Nationwide Children's Hospital Animal Use and Care Committee. *Notch3^+/−^* mice were crossed to produce wild-type and Notch3^−/−^ embryos for analysis. Embryos were fixed in 4% paraformaldehyde overnight at 4°C, embedded in paraffin, and sectioned at 8 μm. For immunohistochemistry, sections were blocked with 10% goat serum and 1% BSA diluted in PBS with 0.5% Triton-X-100 for 1 h at room temperature. Sections were then incubated with primary antibody, anti-Acta2 (1:1,000, Cat: A2547; Sigma), overnight at 4°C, followed by staining with the appropriate Alexa Fluor–conjugated secondary antibody (1:500; Invitrogen) in PBT for 1 h at room temperature, counterstained with 4′,6-diamidino-2-phenylindole (DAPI), and mounted in Vectashield mounting medium (H-1400; Vector Laboratories). Pictures were taken using a fluorescence microscope (1×51; OLYMPUS). Fluorescence of desired vessels was separated out and subtracted from the background to quantify Acta2 intensity. SMA and DAPI costained cells were counted with Cell Count plug-in. Fluorescent images were analyzed with ImageJ software by an observer blinded to sample genotype. Arch measurements of Acta2 staining in mouse sections were normalized to corresponding paired dorsal aortas on the same section to correct for intersample variations in staining intensity.

### Statistical analysis

Results are presented as mean±SD of three independent experiments unless otherwise stated. Statistical analysis was performed using Student's *t*-test.

## Results

### Expression of Notch pathway components during lineage-specific SMC development

**FIG. 1.**
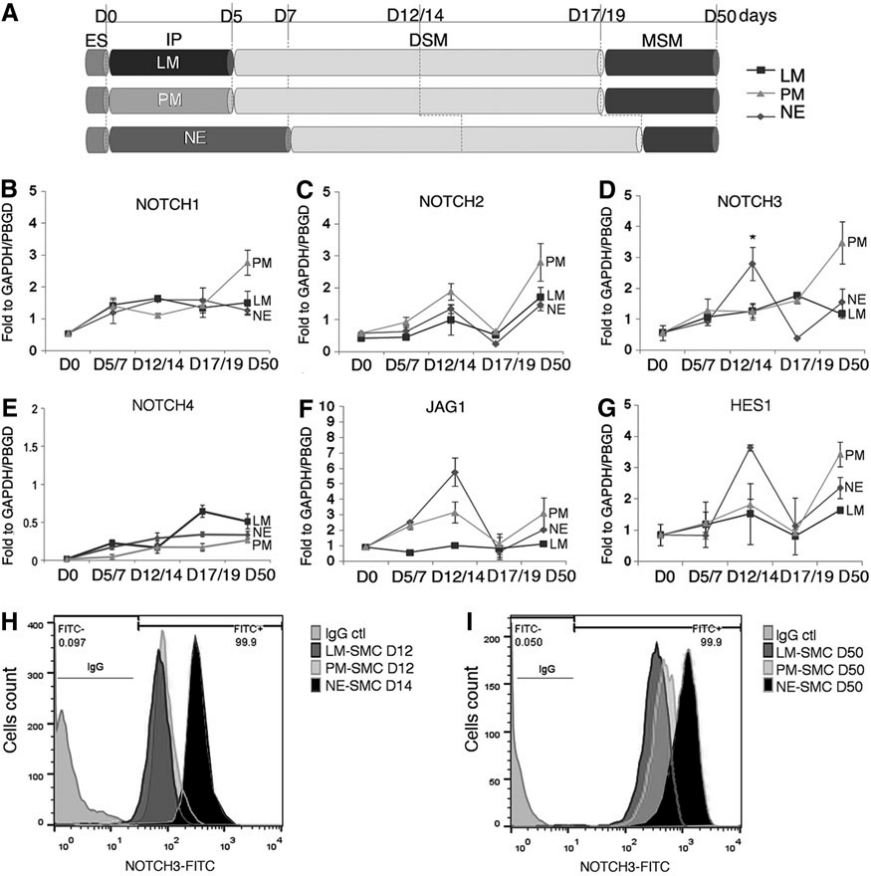
*NOTCH3*, *JAG1*, and *HES1* show a similar expression trend during the differentiation of SMCs of neuroectoderm (NE) origin. **(A)** A schematic representing the timescale of SMC differentiation from hESC to the three different origin-specific subtypes: lateral mesoderm (LM), paraxial mesoderm (PM), and NE. RNA was extracted at the hESC stage (day 0), at the intermediate population stage (day 5/7), at the early SMC differentiation stage (day 12/14), at end of the differentiation protocol (day 17/19), and at the mature stage (day 50) after 30 days of culture in serum-containing media. The transcript levels of Notch receptors **(***NOTCH1*, *NOTCH2*, *NOTCH3*, and *NOTCH4*; **B–E)**, ligands **(***JAG1*; **F)**, and downstream effectors **(***HES1*; **G)** were detected by qRT-PCR. The expression was calculated relative to the housekeeping genes, *GAPDH* and *PBGD*. The *asterisk* indicates a statistically significant difference in the expression of *NOTCH3* in the NE-SMCs in comparison with LM and PM-SMC lineages; *P<0.05. **(H, I)** Flow cytometry analysis demonstrated that NE-SMCs at the early differentiation stage **(**day 14; **H)** were highly positive for NOTCH3 at protein levels (NOTCH3-FITC), while at the mature stage **(**day 50; **I)**, the PM-SMC was more positive for NOTCH3. Values represent mean±SD (*n*=3). DSM, differentiating smooth muscle cells; IP, intermediate populations; MSM, mature smooth muscle cells; NE-SMC, neuroectoderm-derived SMC; qRT-PCR, quantitative real-time polymerase chain reaction; PM-SMC, paraxial mesoderm-derived SMC; SMC, smooth muscle cell.

The H9 hESC line was differentiated into the three early lineages from which most vascular SMCs arise, as we previously described [9]. For NE differentiation, hESCs were incubated with fibroblast growth factor 2 (FGF2, 12 ng/mL) and the activin/nodal inhibitor SB431542 (10 μM) for 7 days to generate the NE intermediate population, which expresses the specific marker *PAX1* (Fig. 1A and Supplementary Fig. S1C). For mesoderm subtype differentiation, we used a combination of FGF2 (20 ng/mL), phosphoinositide 3-kinase (PI3K) inhibitor (LY294002, 10 μM), and bone morphogenetic protein 4 (BMP4, 10 ng/mL) for 36 h to generate an early mesoderm intermediate population. For subsequent specification of LM and PM, the early mesoderm population was respectively incubated for 3 days with FGF2 (20 ng/mL) and LY294002 (10 μM) to generate paraxial mesoderm progenitors expressing the specific marker *TBX6* or with FGF2 (20 ng/mL) and BMP4 (50 ng/mL) to generate a lateral mesoderm intermediate, which expresses *NKX2.5* as a specific marker (Fig. 1A and Supplementary Fig. S1A, B). Further treatment with a combination of PDGF-BB (10 ng/mL) and TGF-β1 (2 ng/mL) for 12 days promoted the differentiation of all the intermediate populations into origin-specific SMCs, which express high levels of specific SM markers, including Calponin (*CNN1*) and SM myosin heavy chain (*MYH11*) (Fig. 1A and Supplementary Fig. S1A–C).

We assessed the RNA expression of the four human notch receptors (*NOTCH1*, *NOTCH2*, *NOTCH3*, and *NOTCH4*), the delta-like (*DLL1*, *DLL3*, and *DLL4*), and jagged (*JAG1* and *JAG2*) ligands, and the downstream effectors, *HES1*, *HEY1*, and *HEY2*, by quantitative RT-PCR at different time points during the differentiation of origin-specific SMC subtypes (Fig. 1B–G and Supplementary Fig. S1D–I). Specifically, RNA levels were measured at day 0 (pluripotent stem cell stage; hESC); at the intermediate population stage, day 5 (D5) for LM and PM and day 7 (D7) for NE; at the intermediate stage of differentiating smooth muscle (DSM) at day 12 (D12) for LM and PM subtypes and at day 14 (D14) for NE lineage; at the end of the differentiation protocol at day 17 (D17) for LM and PM and at day 19 (D19) for NE-SMCs; and finally at the mature smooth muscle stage (MSM; day 50) after a further 30 days of culture in serum-containing media. Notably, at the DSM stage, among the four NOTCH receptors, only NOTCH3 showed a statistically significant difference in expression level between different lineages, with higher expression exclusively in developing NE-SMCs both at RNA and protein levels (Fig. 1D, H). A comparable expression pattern for NOTCH3 was seen in the hiPSC line at RNA level as shown in Supplementary Fig. S2. Similarly Jagged1 (*JAG1*) and Hes-1 (*HES1*) RNA levels increased specifically in the NE-SMC subtype at day 14 (Fig. 1F, G). These data demonstrate a lineage-specific expression pattern for *JAG1*, *HES1*, and *NOTCH3* in the developing SMCs of NE origin and raise the possibility that *JAG1* and *HES1* may be downstream of Notch3 signaling.

Conversely, in mature SMCs after 30 days of culturing in serum-containing medium, the expression of most Notch components, including *NOTCH1*, *NOTCH2*, *NOTCH3*, *JAG1*, *HEY1*, and *HES1*, increased substantially in PM-SMCs (Fig. 1B–D, F, G and Supplementary Fig. S1H). NOTCH3 increased expression was also observed at the protein level by flow cytometric analysis as shown in Fig. 1I. Collectively, these in vitro data suggest that NOTCH3 expression varies according to the lineage and the stage of SMC development.

### NOTCH3 is highly expressed in fetal descending aorta

**FIG. 2.**
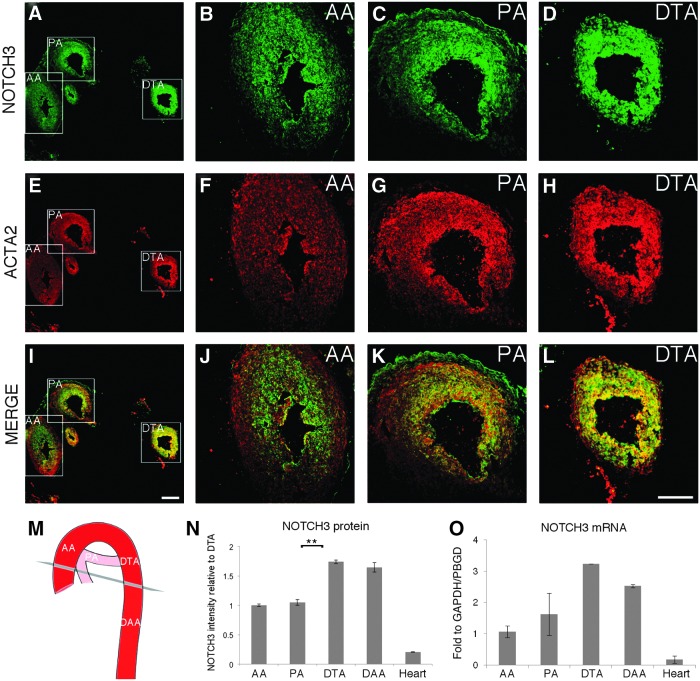
Immunostaining and transcript expression analysis in human fetal aorta shows higher level of NOTCH3 in the descending portion of the aorta. **(A–L)** Sequential sections were taken from cryopreserved tissue of human fetal aorta of 8–9 weeks embryos. The sections were costained with a specific antibody for NOTCH3 **(***green*; **A–D)** and an antibody for ACTA2 **(***red*; **E–H)**. Low-magnification images of a single section show staining for NOTCH3 **(A)**, ACTA2 **(E)**, and merge **(I)** in the ascending aorta (AA), pulmonary artery (PA), and descending thoracic aorta (DTA). High magnification of NOTCH3 staining showed higher intensity in DTA **(D)** than AA **(B)** and PA **(C)**. **(M)** Schematic representing the section orientation and the different aortic regions. **(N)** Quantification of NOTCH3 staining intensity in different aortic regions is expressed relative to the expression in DTA (*n*=3). The *asterisks* indicate statistically significant difference between DTA and PA. **P<0.001. **(O)** RNA from each dissected portion of aorta was extracted and the *NOTCH3* transcript level was quantified by qRT-PCR. Values represent mean±SD (*n*=2). The expression was calculated relative to the housekeeping genes, *GAPDH* and *PBGD*. DAA, descending abdominal aorta. Scale bar=50 μm.

To determine whether our in vitro findings had any physiological relevance, we initially stained sequential sections of human 8–9 week fetal aortas with a specific antibody for NOTCH3 (Fig. 2A–D). The same sections were co-stained with an antibody specific for smooth muscle α-actin (ACTA2) to visualize the layer of SMCs in the aortic wall (Fig. 2E–H). Quantification of NOTCH3 immunostaining revealed a higher expression in the DTA (Fig. 2N) and in the DAA (Fig. 2N and Supplementary Fig. S3A), which are populated by PM-SMCs, in comparison with the ascending aorta (AA), pulmonary artery (PA), and the heart (Supplementary Fig. S3B). In agreement with the protein distribution, *NOTCH3* mRNA levels were higher in the DTA and DAA portions of the aorta (Fig. 2O). Based on these data, we speculate that the in vitro serum matured SMCs (day 50; Fig. 1D, I), which also show higher NOTCH3 expression in PM-SMCs than in the other lineages, are comparable in development with SMCs in the 8–9 week or older fetal aorta.

Taken together, these data strongly suggest that our hESC-derived lineage-specific SMCs have relevance for modeling and predicting in vivo developmental events.

### Mature SMC marker expression is affected by NOTCH3 knockdown

**FIG. 3.**
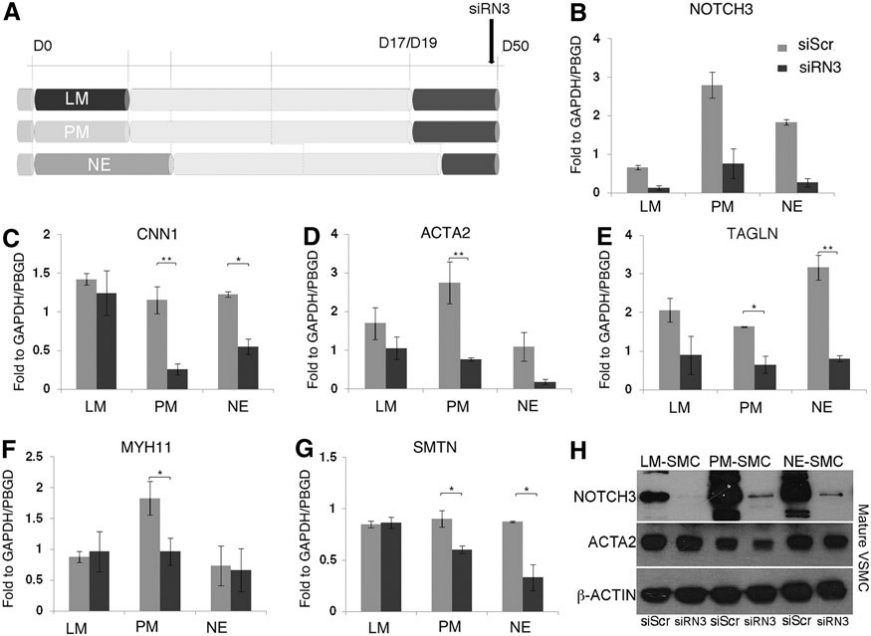
Impaired late SMC differentiation in response to NOTCH3 knockdown in lineages where *NOTCH3* is highly expressed. **(A)** Mature SMCs cultured for 30 days in serum-containing media were transfected with a specific siRNA for *NOTCH3* (siRN3) and with a scrambled siRNA as control (siScr). Cells were harvested after 2 days and transcript levels were quantified by qRT-PCR. **(B)** *NOTCH3* expression was significantly reduced in the three lineages upon siRN3 treatment. *CNN1* **(C)**, *TAGLN* **(E)**, and *SMTN* **(G)** levels were downregulated in PM-SMC and NE-SMC lineages in siRN3 samples. *ACTA2* **(D)** and *MYH11* **(F)** expressions were significantly reduced in PM-SMCs only upon siRN3 treatment. The expression was calculated relative to the housekeeping genes, *GAPDH* and *PBGD*. Values represent mean±SD (*n*=3). The *asterisks* indicate statistically significant differences in comparison with the scrambled siRNA transfected cells; *P<0.05; **P<0.001. **(H)** Western blot analysis showed reduction of ACTA2 in PM-SMC siRN3-treated samples only. β-actin was used as loading control (*n*=2).

To investigate the role of Notch3 in serum matured SMCs, which are comparable to the human fetal aortic SMCs, *NOTCH3* expression was knocked down using a siRNA-mediated approach. A specific siRNA (siRN3) was transfected in fully differentiated MSM (day 50; SMC), which had been cultured in serum-containing media for 1 month (Fig. 3A), the cells were then harvested, and the expression levels for SMC markers were assessed. SiRN3 was successful in knocking down the *NOTCH3* gene expression by ∼70% when compared with the control treated with scrambled siRNA as shown in Fig. 3B.

Interestingly, effective downregulation of *NOTCH3* negatively affected the expression of both early SMC markers, including Calponin (*CNN1*), SM α-actin (*ACTA2*), and SM22 (*TAGLN*; Fig. 3C–E), and late SMC markers, including SM myosin heavy chain (*MYH11*) and Smoothelin (*SMTN*; Fig. 3F, G) in PM-SMCs, while lateral mesoderm-derived SMCs showed no difference from the control. Protein analysis by western blot also confirmed a reduction of ACTA2 in the PM-SMC sample upon siRN3 treatment (Fig. 3H). Similar to PM-SMCs, in SMCs of NE origin, *CNN1*, *TAGLN*, and *SMTN* showed significant decreased RNA levels upon *NOTCH3* knockdown (Fig. 3C, E, G). However, NOTCH3 siRNA treatment had no effect on *MYH11* expression (Fig. 3F) and a nonsignificant effect on *ACTA2* either at RNA or protein levels (Fig. 3D, H) in NE-SMCs. Although it has been shown that both these genes are direct targets of Notch ICD activity, previous studies have not carried out a lineage-specific analysis of Notch induction [24,25]. Our results demonstrate that regulation of SMC markers by Notch3 is critically dependent on cell lineage. Moreover, *JAG1* was downregulated upon Notch3 knockdown exclusively in the NE subtype (Supplementary Fig. S4A). Thus, *JAG1* expression specifically depends on Notch3 function in NE-SMCs and raises the possibility that it in turn activates Notch3. Neither *JAG1* nor *JAG2* expression was affected in PM-SMCs by *NOTCH3* knockdown (Supplementary Fig. S4A, B); however, a significant downregulation of *HEY1* was observed (Supplementary Fig. S4C). From these observations, we conclude that Notch3 is able to control the expression of SM markers in mature SMC lineages of NE and PM origins, although not in lateral mesoderm-derived SMCs. Moreover, the possible positive feedback loop between Notch3 and its ligand, Jagged1, is specific for the NE-SMCs.

### NOTCH3 is required for SMC marker expression during early SMC development from NE in vitro

**FIG. 4.**
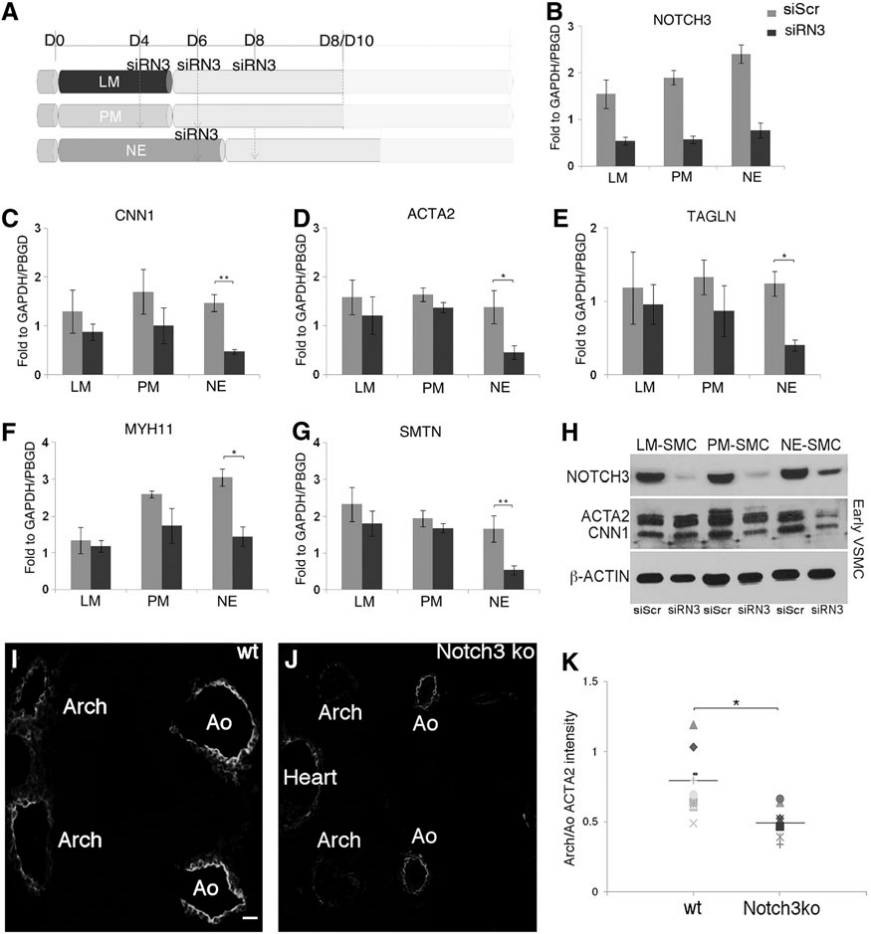
Lineage-specific early NOTCH3 dependence in vitro predicts neural crest phenotype in Notch3-null embryos. **(A)** siRN3 and a siScr as control were transfected twice in intermediate population for LM and PM at days 4 and 6 and for NE at days 6 and 8 as shown in the schematic. Cells were harvested at day 8/10 and transcript levels were quantified by qRT-PCR. **(B)** *NOTCH3* was effectively knocked down in the three lineages upon siRNA treatment. *CNN1* **(C)**, *ACTA2* **(D)**, *TAGLN* **(E)**, *MYH11* **(F)**, and *SMTN* **(G)** were negatively affected by *NOTCH3* knockdown in the SMCs of NE origin. The expression was calculated relative to the housekeeping genes, *GAPDH* and *PBGD*. Values represent mean±SD (*n*=3). The *asterisks* indicate statistically significant differences in comparison with the siScr transfected cells; *P<0.05; **P<0.001. **(H)** Western blot analysis of NOTCH3, ACTA2, and CNN1 in siScr and siRN3 samples. β-actin was used as loading control (*n*=2). **(I, J)** Transverse sections of wt and Notch3^−/−^ embryos at E10.5 were stained for Acta2. **(K)** Comparison of Acta2 staining intensity in the Arch between wt (*n*=10) and Notch3 null (*n*=8) shows significant decrease of expression in the latter (*P<0.05). Ao, dorsal aorta.

We next investigated the role of Notch3 in early SMC development, again using the siRNA-mediated knockdown approach. siRN3 was transfected at day 4 in lateral mesoderm and PM precursors, and then repeated at day 6 (Fig. 4A). Similarly the NE intermediate population was transfected once at day 6 and a second time at day 8 (Fig. 4A). A double transfection was more effective in reducing NOTCH3 at both RNA and protein levels than a single transduction as shown in Supplementary Fig. S5A and B. Figure 4B shows that *NOTCH3* gene expression was reduced by ∼70% following siRN3 treatment. We observed that a range of smooth muscle markers, including early markers (*CNN1*, *ACTA2*, and *TAGLN*; Fig. 4C–E) and late markers (*MYH11* and *SMTN*; Fig. 4F, G), were strongly downregulated upon knockdown of *NOTCH3*, specifically in NE-SMCs. In addition, protein analysis by western blot and immunofluorescence confirmed that the downregulation of both ACTA2 and CNN1 occurs predominantly in NE lineage upon *NOTCH3* knockdown (Fig. 4H and Supplementary Fig. S6C). Furthermore, *JAG1* and *HES1* appear to be negatively affected by *NOTCH3* downregulation in NE-SMCs (Supplementary Fig. S6A, B). This is consistent with our previous observations in mature SMCs, which support a positive feedback mechanism between components of the Notch pathway, which in turn controls the expression of specific SMC markers.

To confirm the importance of Notch3 in regulating the expression of SM markers in the NE-SMC lineage, we used a specific antibody to block exclusively Notch3 activity (AbN3; Supplementary Fig. S7) [22]. The blocking antibody was added to the intermediate populations at day 5 for lateral mesoderm and PM and at day 7 for NE (Supplementary Fig. S7A), and after 7 days the cells were harvested and tested for SM marker expression. Similar to our previous findings using siRNA, a set of SM markers was downregulated only in NE-SMCs after the treatment with AbN3 when compared with control cells treated with an antibody raised against Desmin (AbDesmin; Supplementary Fig. S7B–F). Thus, using two different approaches, we have confirmed that Notch3 activity is critical for the early expression of SM markers exclusively in SMCs of NE origin.

### ACTA2 expression in aortic SMCs is reduced in Notch3-null mice

Although the Notch3-null embryo has not been reported to have a phenotype before birth [23,26], our data reveal a specific requirement for Notch3 in early NE-SMCs. These in vitro findings thus predict a physiological role in SMC development around the aortic arches in the early embryo. Consequently, to validate the in vivo significance of our hESC-derived SMC model, we measured the intensity of SM α-actin (Acta2) expression in SMCs of aortic arch sections of E10.5 Notch3-null mice (Fig. 4J; *n*=8) and compared them with the corresponding sections from wt mice (Fig. 4I; *n*=10). Since our in vitro model predicted that there would be no change in dorsal aorta SM α-actin staining (as this is PM-SMC), then in both Notch3-null and wt sections, arch measurements were normalized to corresponding paired dorsal aortas on the same section to correct for intersample variations in staining intensity. Notch3-null arch sections were found to have a significant decrease in SM α-actin staining, in comparison with the wt (Fig. 4K). Nevertheless, the number of SMCs did not differ between wt and null embryos (expressed as DAPI staining quantification of the SM α-actin positive cells; Supplementary Fig. S8), implying a failure of SMC differentiation in the cells investing the aortic arches. Taken together, these data strongly suggest that our hESC-derived lineage-specific SMCs have relevance for modeling and predicting in vivo developmental events.

## Discussion

Notch signaling is known to be important for vascular development and especially for the regulation of SMC differentiation. However, vascular SMCs are heterogeneous and arise from multiple embryonic origins, and to date there is a paucity of data on whether the embryonic lineage of SMCs influences their response to Notch. Furthermore, there are few or no human developmental studies and it is unclear to what extent data from the mouse models accurately reflect events in the human vasculature. In this study, we have used an in vitro human ESC-based system to investigate the effects of Notch3 on SMCs with distinctive embryological origins. We have also correlated our in vitro findings with observations from selected regions of human fetal aortas with corresponding embryological origins. Importantly, our in vitro model predicted a previously undocumented neural crest-specific phenotype in the developing aorta in the absence of Notch3 signaling, which was verified by observations in Notch3 null-mouse embryos.

Our model of hESC-derived SMCs shows that Notch receptors, ligands, and downstream factors have unique expression patterns in the different origin-specific SMC subtypes. Interestingly, *NOTCH3* was the only NOTCH receptor to show lineage-specific expression changes during SMC development. In addition, *NOTCH3* and its ligand, *JAG1*, share a similar expression trend in developing and mature SMCs. In particular, *NOTCH3* was found to be highly expressed in PM-SMCs, which have been cultured with serum-containing media for 30 days. This stage of our in vitro model corresponded to the physiological expression of *NOTCH3* in the aortic SMC layer of the 8–9 week human fetus, where Notch3 mRNA and protein levels were found to be higher in areas populated by SMCs of somitic origin, such as the DTA and DAA than other aortic regions. These in vitro–in vivo comparisons allow us to begin to estimate the developmental stage and maturity of the hESC-derived SMCs, a common challenge for many types of in vitro-generated tissues in the field of regenerative medicine [27,28].

On the other hand, during early development, we observed that *NOTCH3* and *JAG1* were upregulated exclusively in NE-SMCs, suggesting a specific role for Notch3 in the early development of this subtype. Consequently, to explore the importance of Notch3 in the different SMC subtypes at different developmental stages, we have performed siRNA-mediated knockdown studies in both mature and developing SMCs. Notably, our results show that at the serum maturation stage, Notch3 is required for maintaining the expression of SMC-specific markers in SMCs of PM origin. This is consistent with previous observations in Notch3-null mice, which display SMC defects associated with postnatal maturation in areas of somitic origin, such as the caudal artery of the tail [18,29].

When *NOTCH3* is knocked down at an earlier stage of SMC development, the expression of SMC-specific markers was significantly downregulated exclusively in the NE lineage. This result was further confirmed by inhibiting the NOTCH3 activity with a specific blocking antibody. These studies demonstrate both lineage-specific and developmental stage-specific requirements for Notch3 signaling in SMC development. Importantly, SMC marker expression levels were not totally abolished by *NOTCH3* knockdown in vitro or in the Notch3-null mouse, suggesting that NOTCH3 is not the sole regulator of SMC differentiation and maturation. This confirms the involvement of other signaling pathways, which may include other Notch receptors [17] or NOTCH-independent pathways, in the complex process of SMC development.

Interestingly, previous studies have shown impaired vasoconstriction of the cerebral arteries in knockout mice, suggesting that Notch3 is required for the functional integrity of neural crest-derived vascular SMCs in adult animals [18]. However, no vascular phenotype has been reported in the Notch3-null embryo [23,26], suggesting that vascular defects manifest postnatally. Remarkably, expression of dominant negative mastermind-like in neural crest-derived tissues using a cre-based system did inhibit SMC differentiation around the aortic arches in mouse embryos [30]. However, this method inhibits signaling through all Notch receptors and does not clarify the role of Notch3.

In this study, we have shown for the first time that Notch3 activity is required during early SMC development, and that potentially in the absence of Notch3, the differentiation of NE-SMCs may be already compromised during embryonic development. Importantly, we have confirmed the physiological relevance of our in vitro findings*,* by analyzing the intensity of Acta2 staining in the aorta of E10.5 Notch3-null mice. Notably, Acta2 immunostaining is markedly reduced in the aortic arch, in accordance with our in vitro data, implying that Notch3 has an exclusive role during the early development of SMCs originated from neural crest precursors. Taken together, these findings illustrate the power of our in vitro model for detecting lineage-specific developmental phenotypes that may have been missed in studies on embryos.

In our in vitro model, we also observed a reduction of *JAG1* expression levels in response to Notch3 inhibition in both developing and mature SMCs of NE origin, suggesting the requirement of a Notch-induced positive feedback loop for SMC differentiation, in agreement with previous findings [29,31]. Indeed, this is a common feedback modality for Notch, which also induces another ligand, DLL4, in cardiac development [32,33]. These positive feedback pathways for Notch permit lateral induction and the establishment of a developmental field, in our case for vascular SMCs.

In differentiated SMCs, in addition to its effects on the PM lineage, *NOTCH3* inhibition also reduced the expression of SMC markers in the NE subtype, with the only exception of *MYH11*. The diverse responses to *NOTCH3* knockdown observed in different SMC lineages may be due to the potential redundancy between Notch receptors and ligands, which function synergistically with the SRF/myocardin complex or independently to activate or repress the expression of SMC markers [17,24]. This may also explain why the SMC phenotype is not seen in all the vessels of Notch3-null adult mice and why Acta2 expression is only partially reduced in the developing NE-SMCs, suggesting that other components of the Notch pathway may to some extent compensate for the absence of Notch3 in a lineage-dependent manner.

Ultimately, our findings provide some insight into the Notch receptor–ligand specificity in regulating the differentiation of different SMC lineages and support the functional importance of NOTCH3 in human vascular development. These findings have potential clinical relevance since mutations in *NOTCH3* cause the late-onset genetic vascular condition, Cerebral Autosomal Dominant Arteriopathy with Subcortical Infarcts and Leukoencephalopathy (CADASIL) [34,35]. In the brain, the pathology is characterized by progressive degeneration of SMCs in small arteries and multiple cerebral infarcts. The pathognomonic finding is the accumulation of a granular osmiophilic material, known as GOM, found in vascular SMCs throughout the body and may be detected even in vessels isolated by skin biopsies. Despite the wide distribution of GOM deposition, it is unclear why the vascular complications appear to be limited to the brain. Our hESC-derived SMC model supports a unique role for Notch3 in the development of the NE-SMC lineage, providing the first human evidence of a direct correlation between the lineage-specific requirement for Notch3 in SMC development and the site-specific pattern of pathology in CASADIL syndrome.

In conclusion, our hESC-derived SMC model provides a new and useful tool for studying human SMC development with a high predictive value in vivo. A particular strength of this system is the ability to investigate the role of the embryonic lineage on developmental mechanisms, which may also have relevance for understanding the regional distribution of congenital and acquired vascular diseases.

## Supplementary Material

Supplemental data

Supplemental data

Supplemental data

Supplemental data

Supplemental data

Supplemental data

Supplemental data

Supplemental data

Supplemental data
