# Supplementary material for: Temporal and Embryonic Lineage-Dependent Regulation of Human Vascular SMC Development by NOTCH3
Source: Stem Cells Dev. 2014 Dec 24;24(7):846–56. doi: 10.1089/scd.2014.0520 (PMC4367523; doi:10.1089/scd.2014.0520)
Supplement: Supplemental data [file Supp_Table1.pdf]

Supplementary Table S1. List of Primers Used in Quantitative Real-Time Polymerase Chain Reaction

Gene	*Species*	*Sequences*
NOTCH1	Human	GACGGACCCAACACTTACAC<br>TCAGGCAGAAGCAGAGGTAG
NOTCH2	Human	CACTCGGGGCCTACTCTGTG<br>GATGTCTCCCTCACAACGCT
NOTCH3	Human	ATCGATGACTGTGCCACAGC<br>TTGGCGCCGATAGAGCACTC
NOTCH4	Human	CCAGGTTTCATAGGCCCAGA<br>GGGTCTCACACTCATCCACA
DELTA-1	Human	GAGGGAGGCCTCGTGGA<br>AGACCCGAAGTGCCTTTGTA
DELTA-3	Human	CGGATGCACTCAACAACCT<br>GAAGATGGCAGGTAGCTCAA
DELTA-4	Human	GCATTGTTTACATTGCATCCTG<br>GCAAACCCCAGCAAGAGAC
JAGGED-1	Human	CTATGATGAGGGGGATGCT<br>CGTCCATTCAGGCACTG
JAGGED-2	Human	TGGGATGCCTGGCACA<br>CCGGCAGATGCAGGA
HES-1	Human	AGGCGGACATTCTGGAAATG<br>CGGTACTTCCCCAGCACACTT
HEY1	Human	GGAGAGGCGCCGCTGTAGTTA<br>CAAGGGCGTGCGCGTCAAAGTA
HEY2	Human	ACAGGGGGTAAAGGCTACTTTG<br>CTGCTGCTGCTGCGTTT
CNN1	Human	GTCCACCCTCCTGGCTTT<br>AAACTTGTTGGTGCCCATCT
ACTA2	Human	CACTGTCAGGAATCCTGTGA<br>CAAAGCCGGCCTTACAGA
TAGLN	Human	TCTTTGAAGGCAAAGACATGG<br>TTATGCTCCTGCGCTTTCTT
SMTN	Human	CGGCTGCGCGTGTCTAATCC<br>CTGTGACCTCCAGCAGCTTCCG
MYH11	Human	AGATGGTTCTGAGGAGGAAACG<br>AAAACTGTAGAAAGTTGCTTATTCACT
GAPDH	Human	AACAGCCTCAAGATCATCAGC<br>GGATGATGTTCTGGAGAGCC
PBGD	Human	ATTACCCCGGGAGACTGAAC<br>GGCTGTTGCTTGGACTTCTC
