# Supplementary material for: Temporal and Embryonic Lineage-Dependent Regulation of Human Vascular SMC Development by NOTCH3
Source: Stem Cells Dev. 2014 Dec 24;24(7):846–56. doi: 10.1089/scd.2014.0520 (PMC4367523; doi:10.1089/scd.2014.0520)
Supplement: Supplemental data [file Supp_Fig5.pdf]

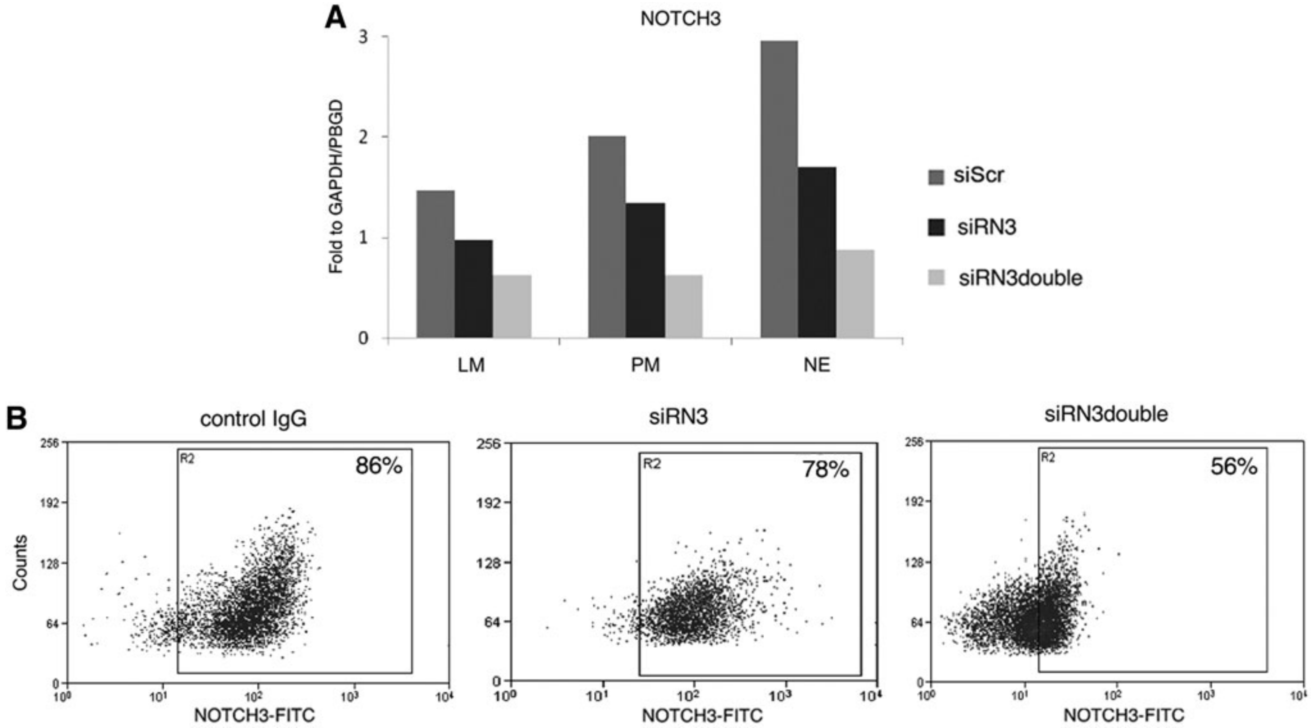

SUPPLEMENTARY FIG. S5. *NOTCH3* knockdown after double transfection with a specific siRNA. **(A)** *NOTCH3* transcript levels were detected by qRT-PCR in SMCs transfected with a control siRNA (siScr), SMCs transfected once with *NOTCH3* siRNA (siRN3), and SMCs transfected twice (siRN3double). **(B)** NOTCH3 protein was detected using a specific anti-NOTCH3 antibody by flow cytometry analysis in control SMCs (control IgG) and SMCs transfected once with siRN3 and twice (siRN3double).
