# Supplementary material for: Temporal and Embryonic Lineage-Dependent Regulation of Human Vascular SMC Development by NOTCH3
Source: Stem Cells Dev. 2014 Dec 24;24(7):846–56. doi: 10.1089/scd.2014.0520 (PMC4367523; doi:10.1089/scd.2014.0520)
Supplement: Supplemental data [file Supp_Fig1.pdf]

Supplementary Data

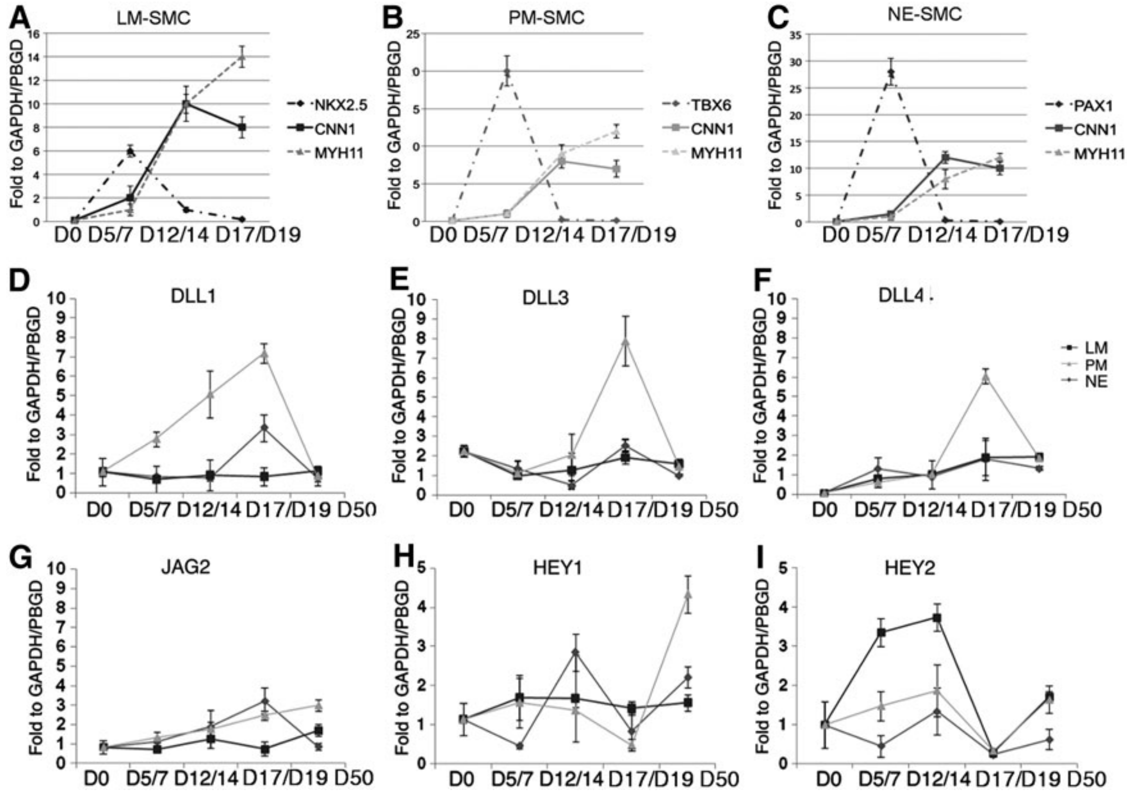

SUPPLEMENTARY FIG. S1. Expression profile of Notch pathway components in distinct SMC lineages. After 5 days of differentiation, specific markers for LM (**A**) and PM (**B**), *NKX2.5* and *TBX6*, respectively, were highly expressed in the intermediate populations, while after 7 days of differentiation, NE intermediate population (**C**) expressed *PAX1* as the specific marker. Further treatment with a combination of PDGF-BB and TGF-β1 for 12 days promoted the differentiation of all the intermediate populations into origin-specific SMCs, which expressed specific SM markers, Calponin (*CNN1*) and SM myosin heavy chain (*MYH11*). Notch ligands, Delta1 (*DLL1*; **D**), Delta3 (*DLL3*; **E**), Delta4 (*DLL4*; **F**), and Jagged2 (*JAG2*; **G**), and downstream effectors, *HEY1* and *HEY2* (**H, I**), expression were detected by qRT-PCR. The expression was calculated relative to the housekeeping genes, *GAPDH* and *PBGD*. Values represent mean ± SD ($n = 3$). LM, lateral mesoderm; NE, neuroectoderm; PM, paraxial mesoderm; qRT-PCR, quantitative real-time polymerase chain reaction; SMC, smooth muscle cell.
