# Supplementary material for: Temporal and Embryonic Lineage-Dependent Regulation of Human Vascular SMC Development by NOTCH3
Source: Stem Cells Dev. 2014 Dec 24;24(7):846–56. doi: 10.1089/scd.2014.0520 (PMC4367523; doi:10.1089/scd.2014.0520)
Supplement: Supplemental data [file Supp_Fig2.pdf]

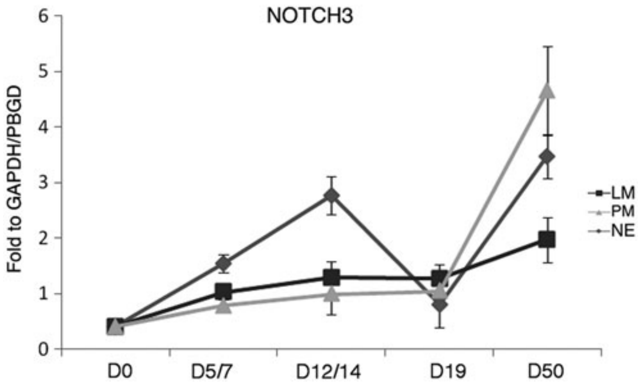

SUPPLEMENTARY FIG. S2. *NOTCH3* expression profile during the differentiation of a human induced pluripotent stem cell line into origin-specific SMC lineages. RNA was extracted at five different time points as follows: at the hESC stage (day 0), at the intermediate population stage (day 5/7), at the intermediate SMC differentiation stage (day 12/14), at end of the differentiation protocol (day 17/19), and at the mature stage (day 50) after 30 days of culture in serum-containing media. The transcript levels of *NOTCH3* were detected by qRT-PCR and the expression was calculated relative to *GAPDH* and *PBGD*. Values represent mean ± SD ($n = 2$).
