# Supplementary material for: Temporal and Embryonic Lineage-Dependent Regulation of Human Vascular SMC Development by NOTCH3
Source: Stem Cells Dev. 2014 Dec 24;24(7):846–56. doi: 10.1089/scd.2014.0520 (PMC4367523; doi:10.1089/scd.2014.0520)
Supplement: Supplemental data [file Supp_Fig3.pdf]

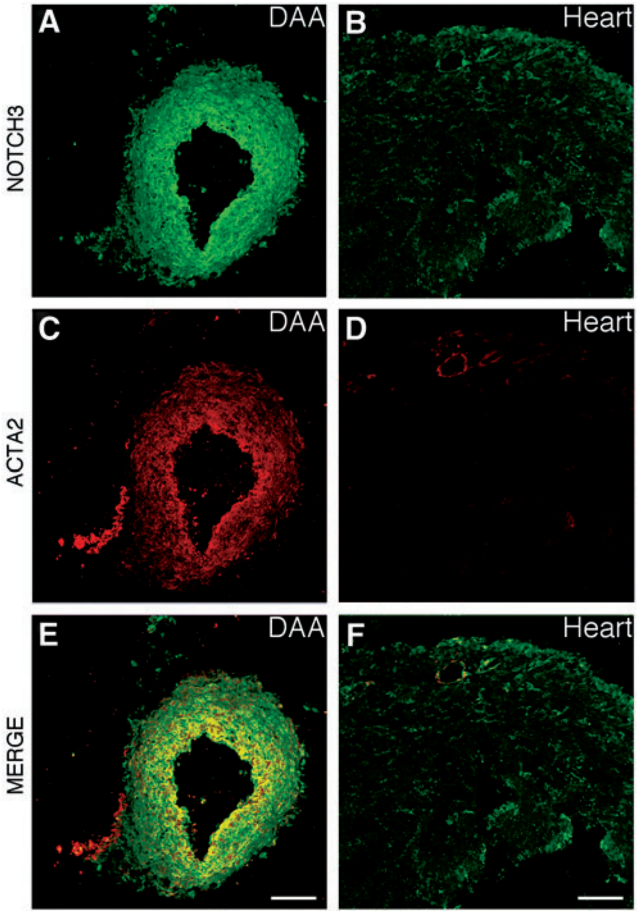

SUPPLEMENTARY FIG. S3. Immunostaining analysis of Notch3 in human fetal abdominal aortic and heart sections. Sections of abdominal aorta (DAA; **A–E)** and heart **(B–F)** from human fetal aorta embryos were stained for NOTCH3 (*green*; **A, B**) and ACTA2 (*red*; **C, D**). **(E, F)** show merged images. Quantification of NOTCH3 staining and RNA levels are shown in Fig. 2. Scale bar = 50 μm.
