# Supplementary material for: Temporal and Embryonic Lineage-Dependent Regulation of Human Vascular SMC Development by NOTCH3
Source: Stem Cells Dev. 2014 Dec 24;24(7):846–56. doi: 10.1089/scd.2014.0520 (PMC4367523; doi:10.1089/scd.2014.0520)
Supplement: Supplemental data [file Supp_Fig4.pdf]

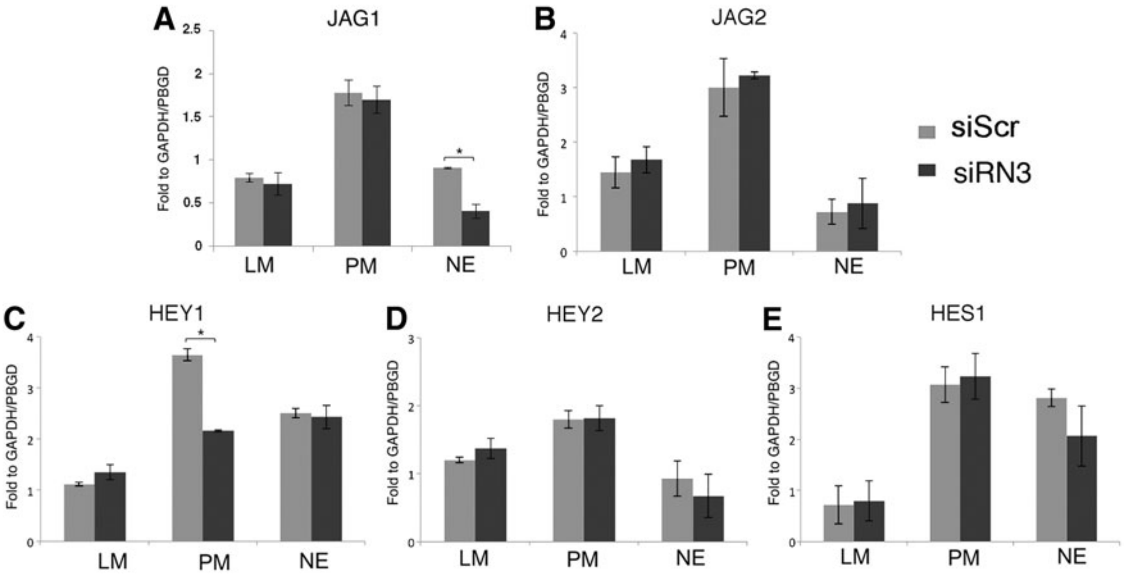

SUPPLEMENTARY FIG. S4. Effects of siRN3 on the expression of Notch ligands, *JAG1* and *JAG2*, and effectors, *HEYs* and *HES1*, in mature SMCs. After being cultured for 30 days in serum-containing media, SMCs were transfected with siRN3 and with a siScr as control. Cells were harvested after 2 days and transcript levels were quantified by qRT-PCR. **(A)** JAG1 expression was downregulated in mature NE-SMCs in response to *NOTCH3* knockdown. **(C)** *HEY1* expression level was negatively affected in PM-SMCs. *JAG2* **(B)**, *HEY2* **(D)**, and *HES1* **(E)** levels were unaffected. The expression was calculated relative to the housekeeping genes, *GAPDH* and *PBGD*. Values represent mean ± SD ($n = 3$). The *asterisks* indicate statistically significant differences in comparison with the scrambled siRNA transfected cells; $^*P < 0.05$. NE-SMC, neuroectoderm-derived SMC; PM-SMC, paraxial mesoderm-derived SMC.
