# Supplementary material for: Temporal and Embryonic Lineage-Dependent Regulation of Human Vascular SMC Development by NOTCH3
Source: Stem Cells Dev. 2014 Dec 24;24(7):846–56. doi: 10.1089/scd.2014.0520 (PMC4367523; doi:10.1089/scd.2014.0520)
Supplement: Supplemental data [file Supp_Fig6.pdf]

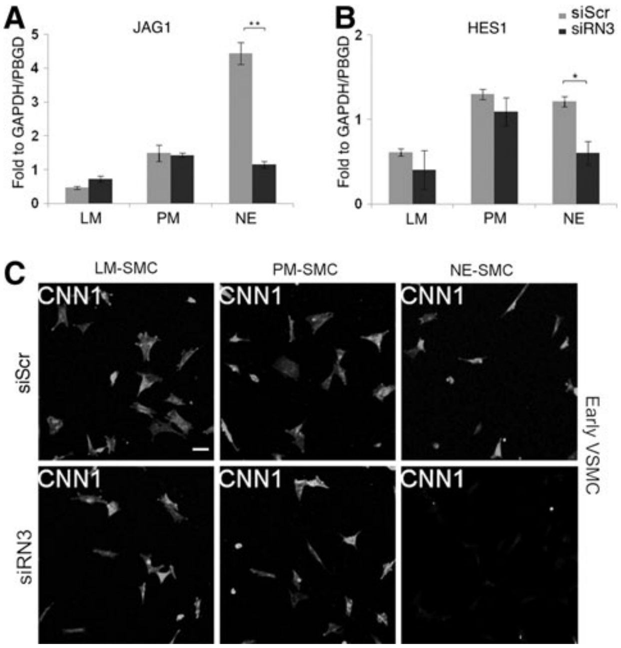

SUPPLEMENTARY FIG. S6. Silencing Notch3 affects *JAG1* **(A)** and *HES1* **(B)** expression in SMCs of NE origin. The expression was calculated relative to *GAPDH* and *PBGD*. Values represent mean ± SD ($n=3$). The asterisks indicate statistically significant differences in comparison with the siScr transfected cells; **P < 0.001. **(C)** Immunostaining for SM early marker, Calponin (CNN1), shows decreased intensity in NE-SMCs upon siRN3 treatment compared with control siScr ($n=2$). Scale bar = 10 μm.
