# Supplementary material for: Temporal and Embryonic Lineage-Dependent Regulation of Human Vascular SMC Development by NOTCH3
Source: Stem Cells Dev. 2014 Dec 24;24(7):846–56. doi: 10.1089/scd.2014.0520 (PMC4367523; doi:10.1089/scd.2014.0520)
Supplement: Supplemental data [file Supp_Fig7.pdf]

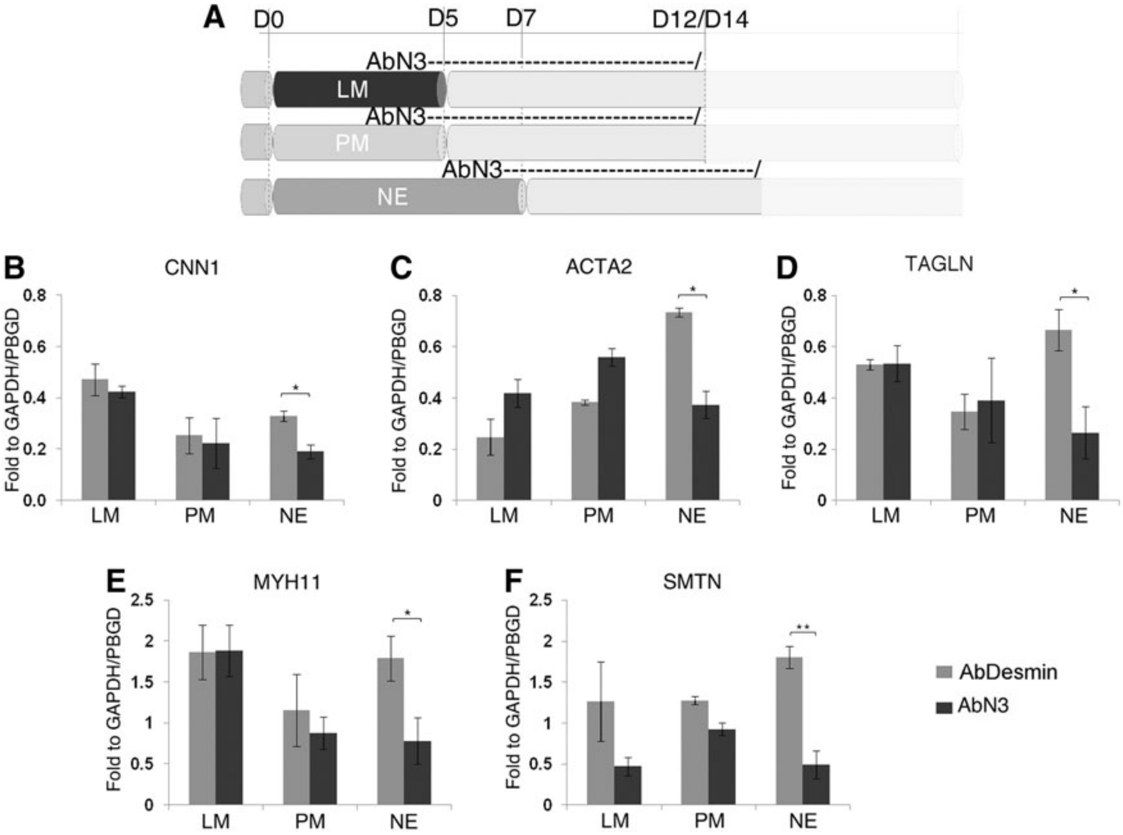

SUPPLEMENTARY FIG. S7. Effect of inhibiting NOTCH3 using a specific blocking antibody during the early differentiation of SMCs. **(A)** A blocking antibody specific for NOTCH3 (AbN3) was added to LM and PM intermediate populations at day 5 and to NE intermediate population at day 7, and cells were harvested after 7 days of incubation as shown in the schematic. Transcript levels were quantified by qRT-PCR in control cells treated with an anti-Desmin antibody (AbDesmin) and with AbN3 antibody. **(B–F)** *CNN1*, *ACTA2*, *TAGLN*, *MYH11*, and *SMTN* expression levels were downregulated specifically in developing NE-SMCs upon treatment with AbN3. The expression was calculated relative to *GAPDH* and *PBGD*. Values represent mean ± SD ($n = 3$). The *asterisks* indicate statistically significant differences in comparison with the AbDesmin cells; $^{*}P < 0.05$; $^{**}P < 0.001$.
