# Supplementary material for: Temporal and Embryonic Lineage-Dependent Regulation of Human Vascular SMC Development by NOTCH3
Source: Stem Cells Dev. 2014 Dec 24;24(7):846–56. doi: 10.1089/scd.2014.0520 (PMC4367523; doi:10.1089/scd.2014.0520)
Supplement: Supplemental data [file Supp_Fig8.pdf]

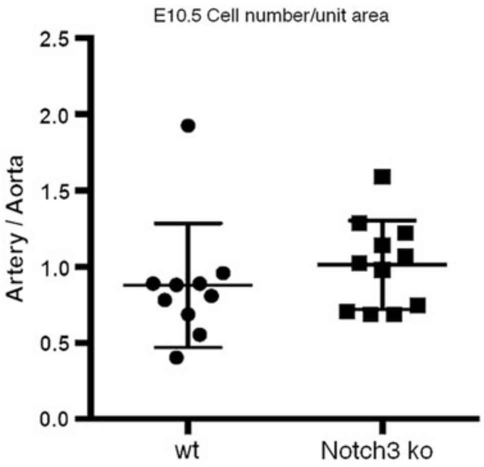

SUPPLEMENTARY FIG. S8. Quantification of SMC number in E10.5 wt and Notch3$^{-/-}$ embryos by DAPI staining. DAPI, 4′,6-diamidino-2-phenylindole.
